# Dynamic Co‐Evolution of C‐Reactive Protein and Albumin in Early Sepsis: A Group‐Based Dual‐Trajectory Analysis for Risk Stratification

**DOI:** 10.1111/jcmm.71402

**Published:** 2026-10-14

**Authors:** Tengfei Chen, Shuwen Zhang, Maoyu Ding, Yumei Yang, Qinxiang Yang, Wei Guo, Xiaolong Xu, Qingquan Liu

**Affiliations:** ^1^ Beijing Hospital of Traditional Chinese Medicine Capital Medical University Beijing China; ^2^ Beijing Institute of Traditional Chinese Medicine Beijing China; ^3^ Beijing Key Laboratory of Innovative Research on Removing Stasis and Detoxification Theory in Infectious Diseases (BZ‐2024‐078) Beijing China; ^4^ Beijing University of Chinese Medicine Beijing China

## Abstract

The coupled evolution of inflammation and metabolic dysfunction during early sepsis is incompletely characterized. Although C‐reactive protein (CRP) and albumin (ALB) are established markers of these processes, their joint longitudinal patterns have rarely been examined. We used group‐based dual‐trajectory modelling (GBDTM) to identify distinct coevolutionary patterns of CRP and ALB and to evaluate their associations with mortality in patients with sepsis. This retrospective observational study included patients admitted to the ICU of Beijing Hospital of Traditional Chinese Medicine, Capital Medical University, between June 2014 and March 2025. All included adults met the Sepsis‐3 criteria. Among 462 patients with sepsis, serial CRP and ALB measurements obtained on days 1, 3, 5, 7 and 14 after ICU admission were analysed using GBDTM. The selected three‐class model showed acceptable classification quality (entropy = 0.80; class‐specific average posterior probabilities [AvePPs] = 0.84–0.92) and identified slow‐decreasing (79.65%), rapid‐decreasing (10.39%) and increasing (9.96%) inflammatory phenotypes. The 28‐day mortality rate was highest in the increasing group (52.2% vs. 32.6% and 33.3%; log‐rank *p* = 0.034). In the fully adjusted Cox model, the increasing trajectory was associated with 28‐day mortality (HR 1.943, 95% CI: 1.202–3.140; *p* = 0.007) and 60‐day mortality (HR 1.946, 95% CI: 1.218–3.108; *p* = 0.005). Parsimonious models adjusted for age, sex, and SOFA or the Acute Physiology and Chronic Health Evaluation II (APACHE II) score yielded broadly similar estimates. Joint CRP‐ALB trajectories identify clinically interpretable dynamic phenotypes during early sepsis. These findings support the joint trajectory as a hypothesis‐generating, complementary phenotyping marker that requires external prospective validation.

**Abbreviations**
AGI: acute gastrointestinal injury
AIC: Akaike information criterion
ALB: albumin
ALP: alkaline phosphatase
ALT: alanine aminotransferase
ANOVA: analysis of variance
APACHE II: acute physiology and chronic health evaluation II
APTT: activated partial thromboplastin time
AST: aspartate aminotransferase
AUC: area under the curve
AvePP: average posterior probability
BA: bile acids
BIC: Bayesian information criterion
BNP: B‐type natriuretic peptide
BUN: blood urea nitrogen
CI: confidence interval
CK: creatine kinase
CKMB: creatine kinase myocardial band
Cr: creatinine
CRP: C‐reactive protein
DB: direct bilirubin
DBP: diastolic blood pressure
DD: D‐dimer
eGFR: estimated glomerular filtration rate
FDP: fibrin degradation products
FIB: fibrinogen
FiO2: fraction of inspired oxygen
GBDTM: group‐based dual‐trajectory modelling
GCS: Glasgow Coma Scale
GGT: gamma‐glutamyl transferase
Hct: haematocrit
Hgb: haemoglobin
HR: hazard ratio
ICU: intensive care unit
IL‐6: interleukin‐6
K: potassium
LAAPD: left atrial anteroposterior diameter
LASSO: least absolute shrinkage and selection operator
LDH: lactate dehydrogenase
LVEDD: left ventricular end‐diastolic diameter
LVEF: left ventricular ejection fraction
M: median
MAP: mean arterial pressure
MIMIC: medical information mart for intensive care
Na: sodium
NEU: neutrophils
PaO2: partial pressure of oxygen in arterial blood
PCT: procalcitonin
PLT: platelet count
PT: prothrombin time
Q1: 1st quartile
Q3: 3rd quartile
RBC: red blood cell count
ROC: receiver operating characteristic
SBP: systolic blood pressure
SD: standard deviation
Sepsis‐3: The Third International Consensus Definitions for Sepsis and Septic Shock
SIRS: systemic inflammatory response syndrome
SOFA: Sequential Organ Failure Assessment
TB: total bilirubin
TNI: troponin I
TT: thrombin time
UA: uric acid
WBC: white blood cell count

## 1 Introduction

Sepsis is life‐threatening organ dysfunction caused by a dysregulated host response to infection and remains a major cause of death in intensive care [1, 2, 3]. Its marked clinical heterogeneity reflects divergent immune‐inflammatory and metabolic responses and limits uniform prognostic and therapeutic approaches [4, 5]. Current approaches to sepsis risk stratification rely heavily on measurements obtained at isolated time points and may therefore miss non‐linear changes in the host response during the early phase of illness. This limitation creates a need for longitudinal phenotyping methods that characterize disease dynamics while preserving clinically interpretable patterns.

C‐reactive protein (CRP) and albumin (ALB) are two well‐established and readily accessible biomarkers in sepsis, both primarily synthesized in the liver and closely linked in the host response to infection [6, 7, 8]. As a prototypic positive acute‐phase reactant, CRP is upregulated in response to pro‐inflammatory cytokines—particularly interleukin‐6 (IL‐6)—and serves as a robust indicator of systemic inflammatory activity. In contrast, ALB is a negative acute‐phase protein, and its serum concentration decreases in response to inflammation. Accordingly, the degree of hypoalbuminemia in critically ill patients correlates with the intensity of the inflammatory response triggered by infection [9, 10]. Beyond reflecting nutritional status and hepatic synthetic function, ALB also serves as an indicator of inflammatory burden and vascular permeability [11]. Hypoalbuminemia has been consistently associated with adverse outcomes in critically ill populations [11, 12]. Notably, emerging evidence suggests that composite indices integrating CRP and ALB (e.g., CRP‐to‐ALB ratio and the CALLY index) outperform single biomarkers in the diagnosis and prognostication of sepsis, underscoring the interplay between inflammatory activation and metabolic reserve in determining clinical outcomes [13, 14].

Most studies of CRP and ALB in sepsis have examined static values, composite ratios or separate trajectories of a single biomarker, thereby overlooking their coordinated temporal behaviour. Public critical‐care databases can support external validation, but serial CRP and ALB, especially CRP measurements at standardized time points may be incomplete or irregular [15]. Our single‐center cohort contained repeated CRP and ALB measurements at five prespecified time points (days 1, 3, 5, 7 and 14), enabling analysis of their joint evolution. GBDTM extends conventional group‐based trajectory modelling by jointly modelling correlated longitudinal markers and identifying clinically interpretable subphenotypes [16]. To our knowledge, joint CRP‐ALB trajectories have not previously been characterized in early sepsis. We therefore aimed to describe their temporal patterns and evaluate their associations with 28‐day and 60‐day all‐cause mortality.

## 2 Materials and Methods

### 2.1 Study Design and Participants

This retrospective cohort study was conducted in the Department of Critical Care Medicine at Beijing Hospital of Traditional Chinese Medicine, Capital Medical University. Adults admitted to the ICU between June 2014 and March 2025 were screened. Eligible patients were aged 18 years or older and met the Sepsis‐3 criteria. Patients were excluded if ICU stay was shorter than 24 h, data essential for eligibility or outcome ascertainment were unavailable, or fewer than two CRP or ALB measurements were available for trajectory modelling. A total of 462 patients were included (Figure 1).

**FIGURE 1:**
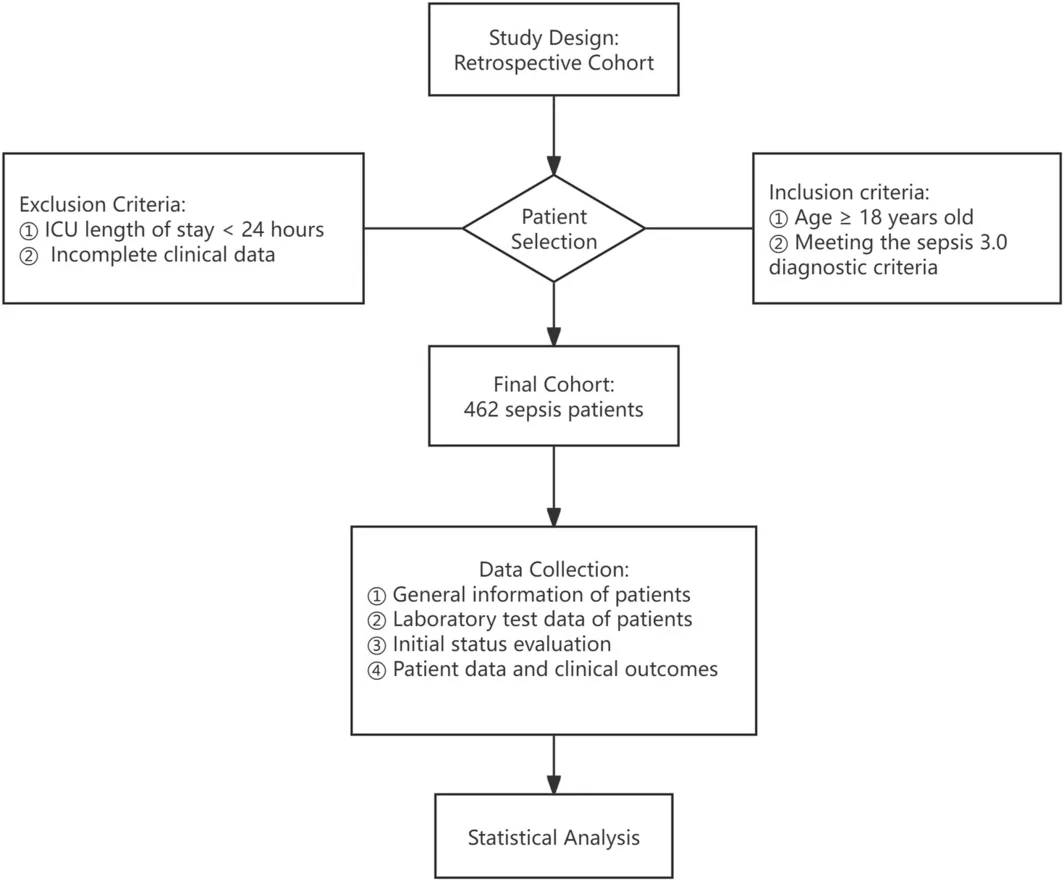
Flowchart of enrolment.

### 2.2 Data Collection and Variable Selection

Demographic and clinical data, including vital signs, comorbidities, infection sites, treatments and laboratory measurements obtained within 24 h of ICU admission, were extracted from electronic medical records. Serum CRP and ALB were recorded on days 1, 3, 5, 7 and 14 after ICU admission. Missing non‐essential baseline covariates were imputed using the random forest‐based missForest algorithm in R.

### 2.3 Statistical Analysis

#### 2.3.1 Group‐Based Dual‐Trajectory Modelling

GBDTM was used to identify latent subgroups with similar longitudinal CRP and ALB patterns. Analyses were performed using the lcmm package in R (version 4.5.1). Linear, quadratic and cubic time functions were evaluated across solutions containing one to six latent classes. Model selection considered log likelihood, Akaike information criterion (AIC), Bayesian information criterion (BIC), entropy (values > 0.70 indicating adequate separation), class‐specific average posterior probabilities (AvePP; values ≥ 0.70 indicating acceptable assignment), minimum class prevalence of 5%, parsimony and clinical interpretability. These criteria follow established trajectory‐modelling recommendations and recent critical‐care applications [17, 18, 19, 20].

#### 2.3.2 Survival and Multivariable Analysis

The primary outcome was 28‐day all‐cause mortality, and the secondary outcome was 60‐day all‐cause mortality. Baseline characteristics were compared using analysis of variance, the Kruskal‐Wallis test, the chi‐square test or Fisher's exact test, as appropriate. Kaplan–Meier curves were compared using the log‐rank test. Candidate variables for least absolute shrinkage and selection operator (LASSO) Cox regression were chosen a priori from clinically relevant demographic, physiological, infection‐related, organ‐function, treatment and laboratory domains; the complete candidate set and rationale are provided in Table S10. Ten‐fold cross‐validation was used to select the regularization parameter. The proportional hazards assumption was assessed for individual covariates and globally using scaled Schoenfeld residuals. All tests were two‐sided, with *p* < 0.05 indicating statistical significance.

#### 2.3.3 Sensitivity and Model‐Performance Analyses

Sensitivity analyses included: (1) separate fully adjusted models using CRP‐only, ALB‐only or joint CRP‐ALB trajectory membership; (2) two‐ and four‐class joint‐trajectory solutions; (3) parsimonious models adjusted for age, sex and either SOFA or APACHE II; (4) comparison of C‐indexes for severity‐score models with and without trajectory membership using compareC; and (5) assessment of variance inflation factors (VIFs), followed by refitting after excluding RBC. Because the single‐marker and joint latent‐class models were non‐nested, their fit was compared descriptively using AIC and Harrell's C‐index rather than pairwise likelihood‐ratio tests.

### 2.4 Ethics Statement

This study was approved by the Ethics Committee of Beijing Hospital of Traditional Chinese Medicine (Approval No. 2024BL02099). Written informed consent was obtained from all participants in accordance with institutional and international ethical standards.

## 3 Results

### 3.1 Identification of Joint Trajectories

GBDTM was performed in 462 patients. Although AIC and BIC decreased as the number of classes increased, improvement was accompanied by progressively smaller classes (Table 1). The selected three‐class solution had an entropy of 0.80 and class‐specific AvePPs of 0.84, 0.87 and 0.92. All classes exceeded the prespecified 5% prevalence threshold; the smallest class contained 46 patients (9.96%). In contrast, the four‐class solution contained a class of 18 patients (3.90%), and the five‐ and six‐class solutions contained still smaller classes. Considering classification quality, class size, parsimony and clinical interpretability, the three‐class model was selected. It identified slow‐decreasing (*n* = 368, 79.65%), rapid‐decreasing (*n* = 48, 10.39%) and increasing (*n* = 46, 9.96%) CRP‐ALB phenotypes (Figure 2).

**TABLE 1:** Comparison of fit indices for the CRP–ALB joint trajectory models.

Fit indices	Group number					
	1	2	3	4	5	6
Log likelihood	−17252.94	−17192.17	−17138.09	−17105.25	−17079.31	−17063.18
AIC	34525.87	34412.34	34312.18	34254.50	34210.63	34186.36
BIC	34567.23	34470.24	34386.62	34345.48	34318.15	34310.43
Entropy	1	0.77	0.80	0.81	0.82	0.82
Class proportion	Class 1, 462/100%	Class 1, 74/16.02%	Class 1, 46/9.96%	Class 1, 30/6.49%	Class 1, 22/4.76%	Class 1, 6/1.30%
		Class 2, 388/83.98%	Class 2, 48/10.39%	Class 2, 52/11.26%	Class 2, 44/9.52%	Class 2, 41/8.87%
			Class 3, 368/79.65%	Class 3, 362/78.35%	Class 3, 339/73.38%	Class 3, 333/72.08%
				Class 4, 18/3.90%	Class 4, 46/9.96%	Class 4, 26/5.63%
					Class 5, 11/2.38%	Class 5, 44/9.52%
						Class 6, 12/2.60%
AvePP	Class 1, 1	Class 1, 0.86	Class 1, 0.84	Class 1, 0.84	Class 1, 0.86	Class 1, 0.91
		Class 2, 0.94	Class 2, 0.87	Class 2, 0.88	Class 2, 0.81	Class 2, 0.82
			Class 3, 0.92	Class 3, 0.90	Class 3, 0.91	Class 3, 0.89
				Class 4, 0.92	Class 4, 0.81	Class 4, 0.78
					Class 5, 0.94	Class 5, 0.83
						Class 6, 0.95

Abbreviations: AIC, Akaike Information Criterion; AvePP, Average Posterior Probability; BIC, Bayesian Information Criterion.

**FIGURE 2:**
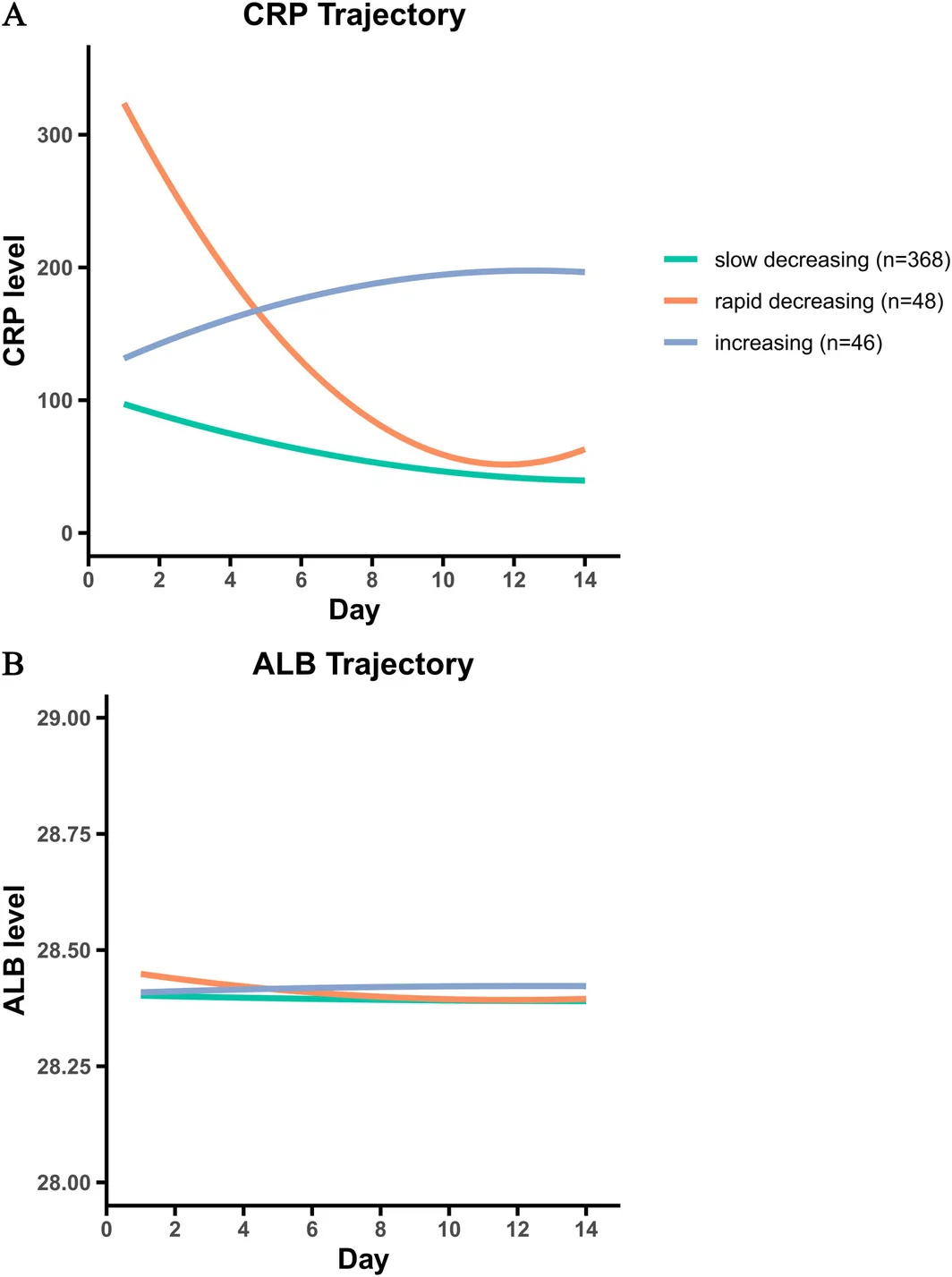
Joint CRP‐ALB trajectories during the first 14 days after ICU admission. (A) Estimated CRP and (B) ALB trajectories for the slow‐decreasing (*n* = 368), rapid‐decreasing (*n* = 48) and increasing (*n* = 46) groups.

### 3.2 Baseline Characteristics

The mean age was 76.29 ± 13.54 years, and 60.8% of patients were male. The increasing group had a lower prevalence of skin and soft‐tissue infection (4.3% vs. 12.2% and 22.9%; *p* = 0.023), while distributions of acute gastrointestinal injury grade (*p* = 0.015), pH (*p* = 0.036) and hemofiltration duration (*p* = 0.019) also differed across groups (Table 2).

**TABLE 2:** Baseline characteristics of the CRP–ALB joint trajectory groups.

Characteristics	Total (*n* = 462)	Dual‐trajectory goup			*p*
		Slow decreasing (*n* = 368)	Rapid decreasing (*n* = 48)	Increasing (*n* = 46)	
Gender, *n*(%)					
Male	281 (60.8)	223 (60.6)	27 (56.2)	31 (67.4)	0.532
Female	181 (39.2)	145 (39.4)	21 (43.8)	15 (32.6)	
Age (year) (mean [SD])	76.29 (13.54)	76.80 (13.69)	76.25 (10.22)	72.26 (14.93)	0.100
Heart rate (/Min), M (Q_1_, Q_3_)	90.50 (78.00, 108.00)	90.50 (78.00, 108.00)	93.00 (82.00, 111.00)	89.00 (83.00, 106.75)	0.285
Respiratory system infection, *n* (%)					
No	88 (19.0)	75 (20.4)	8 (16.7)	5 (10.9)	0.273
Yes	374 (81.0)	293 (79.6)	40 (83.3)	41 (89.1)	
Haematological system infection, *n* (%)					
No	334 (72.3)	271 (73.6)	33 (68.8)	30 (65.2)	0.410
Yes	128 (27.7)	97 (26.4)	15 (31.2)	16 (34.8)	
Digestive system infection, *n* (%)					
No	382 (82.7)	304 (82.6)	39 (81.2)	39 (84.8)	0.899
Yes	80 (17.3)	64 (17.4)	9 (18.8)	7 (15.2)	
Infection of the skin and soft tissues, *n* (%)					
No	404 (87.4)	323 (87.8)	37 (77.1)	44 (95.7)	0.023
Yes	58 (12.6)	45 (12.2)	11 (22.9)	2 (4.3)	
ICU stay (day), M (Q_1_, Q_3_)	14.00 (7.00, 22.00)	14.00 (7.00, 22.00)	11.00 (6.75, 20.00)	14.00 (4.00, 22.50)	0.632
Hospital stay (day), M (Q_1_, Q_3_)	23.00 (12.00, 41.00)	24.00 (12.00, 41.00)	19.00 (10.75, 46.00)	23.50 (13.25, 39.75)	0.798
Invasive ventilator time (h), M (Q_1_, Q_3_)	9.00 (0.00, 149.75)	9.50 (0.00, 147.25)	18.00 (0.00, 122.25)	8.50 (0.00, 178.50)	0.942
Hemofiltration (h), M (Q_1_, Q_3_)	0.00 (0.00, 14.00)	0.00 (0.00, 1.25)	0.00 (0.00, 51.75)	0.00 (0.00, 89.25)	0.019
MAP (mmHg), M (Q_1_, Q_3_)	87.00 (70.42, 98.00)	87.00 (72.00, 100.00)	79.00 (67.75, 98.00)	86.00 (74.00, 93.50)	0.397
AGI, *n* (%)					
0	5 (1.1)	3 (0.8)	2 (4.2)	0 (0.0)	0.015
1	11 (2.4)	6 (1.6)	1 (2.1)	4 (8.7)	
2	29 (6.3)	26 (7.1)	1 (2.1)	2 (4.3)	
3	140 (30.3)	106 (28.8)	20 (41.7)	14 (30.4)	
4	277 (60.0)	227 (61.7)	24 (50.0)	26 (56.5)	
PH, M (Q_1_, Q_3_)	7.43 (7.34, 7.47)	7.43 (7.34, 7.47)	7.40 (7.34, 7.46)	7.45 (7.38, 7.49)	0.036
PaO2 (mmHg), M (Q_1_, Q_3_)	108.00 (80.00, 143.00)	105.00 (80.00, 140.00)	115.50 (80.22, 158.25)	123.50 (80.30, 146.00)	0.390
TB (*μ*mol/L), M (Q_1_, Q_3_)	14.95 (8.72, 26.70)	14.70 (8.70, 26.70)	19.20 (10.23, 26.70)	18.45 (8.38, 26.02)	0.651
Bile acids (*μ*mol/L), M (Q_1_, Q_3_)	5.40 (2.90, 10.90)	5.30 (2.88, 10.90)	6.85 (3.15, 10.90)	5.70 (3.15, 10.90)	0.831
APTT (s), M (Q_1_, Q_3_)	33.10 (29.22, 38.68)	33.25 (29.30, 38.80)	32.30 (29.05, 35.78)	34.30 (29.75, 39.33)	0.490
FDP (mg/L), M (Q_1_, Q_3_)	10.65 (6.12, 21.65)	10.55 (6.00, 21.22)	13.50 (6.38, 22.68)	10.10 (6.72, 23.28)	0.477
BUN (mmol/L), M (Q_1_, Q_3_)	11.30 (6.43, 20.92)	10.60 (6.38, 19.55)	15.90 (7.20, 24.13)	13.20 (8.32, 23.05)	0.104
BNP (ng/L), M (Q_1_, Q_3_)	557.50 (227.00, 1799.67)	539.50 (233.00, 1884.25)	593.00 (198.50, 1557.75)	670.50 (365.75, 1783.00)	0.790
CKMB (ng/ml), M (Q_1_, Q_3_)	2.90 (1.20, 8.90)	2.60 (1.10, 9.57)	4.55 (1.37, 8.45)	3.95 (1.92, 8.67)	0.211
LAAPD (cm), M (Q_1_, Q_3_)	3.50 (3.20, 3.80)	3.50 (3.20, 3.80)	3.45 (3.30, 3.70)	3.45 (3.20, 3.80)	0.871
LVEF, M (Q_1_, Q_3_)	0.61 (0.58, 0.63)	0.60 (0.58, 0.63)	0.62 (0.59, 0.65)	0.60 (0.58, 0.63)	0.409
RBC (*10^12^/L) (mean [SD])	3.17 (0.87)	3.18 (0.87)	3.21 (0.84)	3.05 (0.90)	0.596
Hgb (g/L) (mean [SD])	94.56 (26.26)	94.91 (25.63)	96.10 (29.62)	90.15 (27.71)	0.466
GCS, M (Q_1_, Q_3_)	6.00 (3.00, 14.00)	6.50 (3.00, 14.00)	6.00 (3.00, 13.25)	7.50 (3.00, 15.00)	0.713
Outcome 28 day, *n* (%)					
0 = Life	302 (65.4)	248 (67.4)	32 (66.7)	22 (47.8)	0.031
1 = Death	160 (34.6)	120 (32.6)	16 (33.3)	24 (52.2)	
Outcome 60 day, *n* (%)					
0 = Life	286 (61.9)	234 (63.6)	31 (64.6)	21 (45.7)	0.057
1 = Death	176 (38.1)	134 (36.4)	17 (35.4)	25 (54.3)	

*Note:* The main baseline table was shortened to focus on variables most relevant to the primary analysis; the complete set of baseline characteristics is provided in Table S14.

Abbreviations: M, median; Q1, first quartile; Q3, third quartile; SD, standard deviation.

### 3.3 Survival Analysis

The overall 28‐day mortality rate was 34.6%. Kaplan–Meier analysis demonstrated differences in survival across the three trajectory groups (log‐rank *p* = 0.034 for 28‐day mortality and *p* = 0.049 for 60‐day mortality) (Figure 3). The increasing group had the highest 28‐day mortality rate (52.2%), compared with the slow‐decreasing (32.6%) and rapid‐decreasing (33.3%) groups.

**FIGURE 3:**
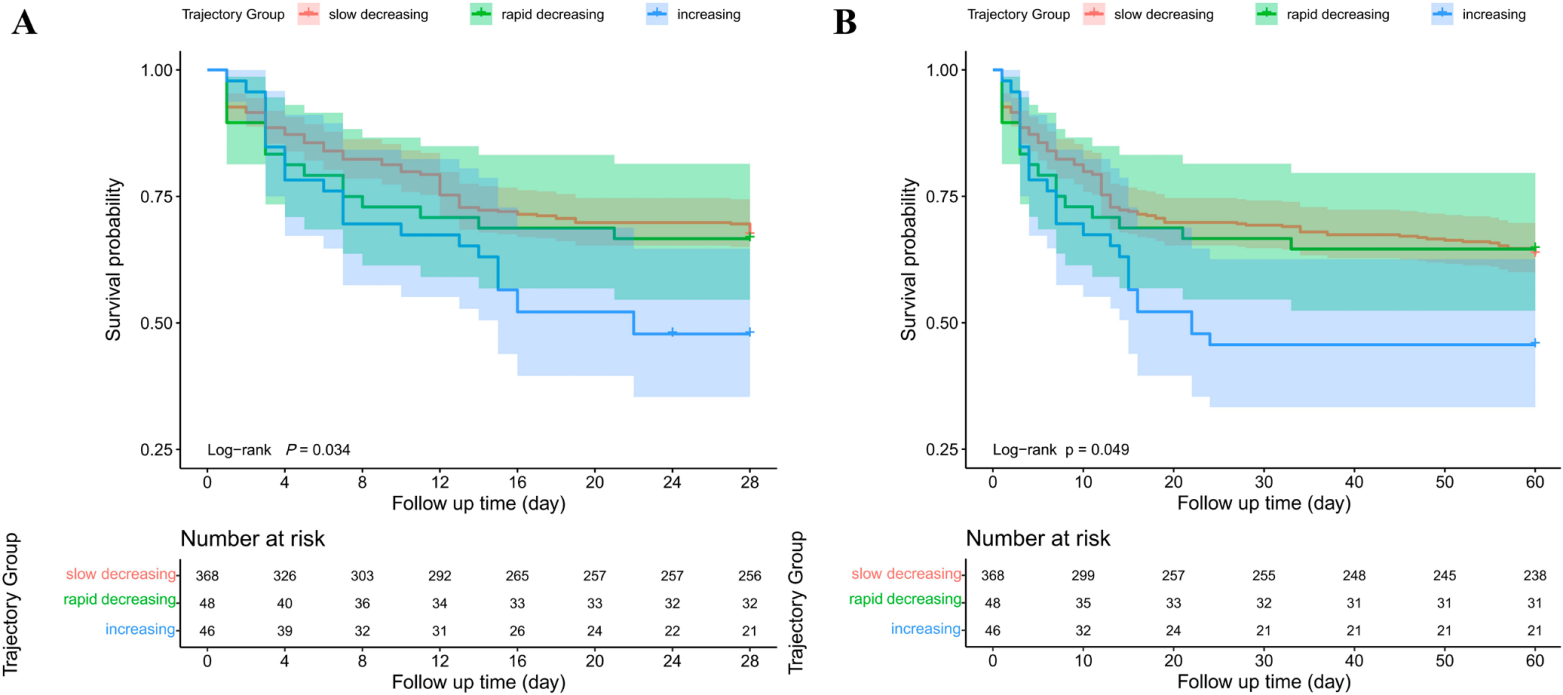
Kaplan–Meier survival curves according to CRP–ALB joint trajectory groups. (A) 28‐day mortality; (B) 60‐day mortality.

### 3.4 Independent Prognostic Value of Dual Trajectories

LASSO selected 27 adjustment covariates in addition to trajectory membership at λ = 0.025 (Figure 4); the complete candidate‐variable inventory and the final adjustment set are provided in Tables S10 and S11. In the fully adjusted model, the increasing trajectory remained associated with 28‐day mortality (HR 1.943, 95% CI: 1.202–3.140; *p* = 0.007) and 60‐day mortality (HR 1.946, 95% CI: 1.218–3.108; *p* = 0.005) relative to the slow‐decreasing group (Table 3). The similar effect estimates observed for 28 and 60‐day mortality indicate that the association between the increasing‐trajectory group and mortality was broadly consistent across the two short‐term follow‐up periods. Scaled Schoenfeld‐residual tests provided no evidence against proportional hazards: the global *p* values were 0.058 for the 28‐day model and 0.683 for the 60‐day model, and all variable‐specific *p* values exceeded 0.05 (Figures S1 and S2).

**FIGURE 4:**
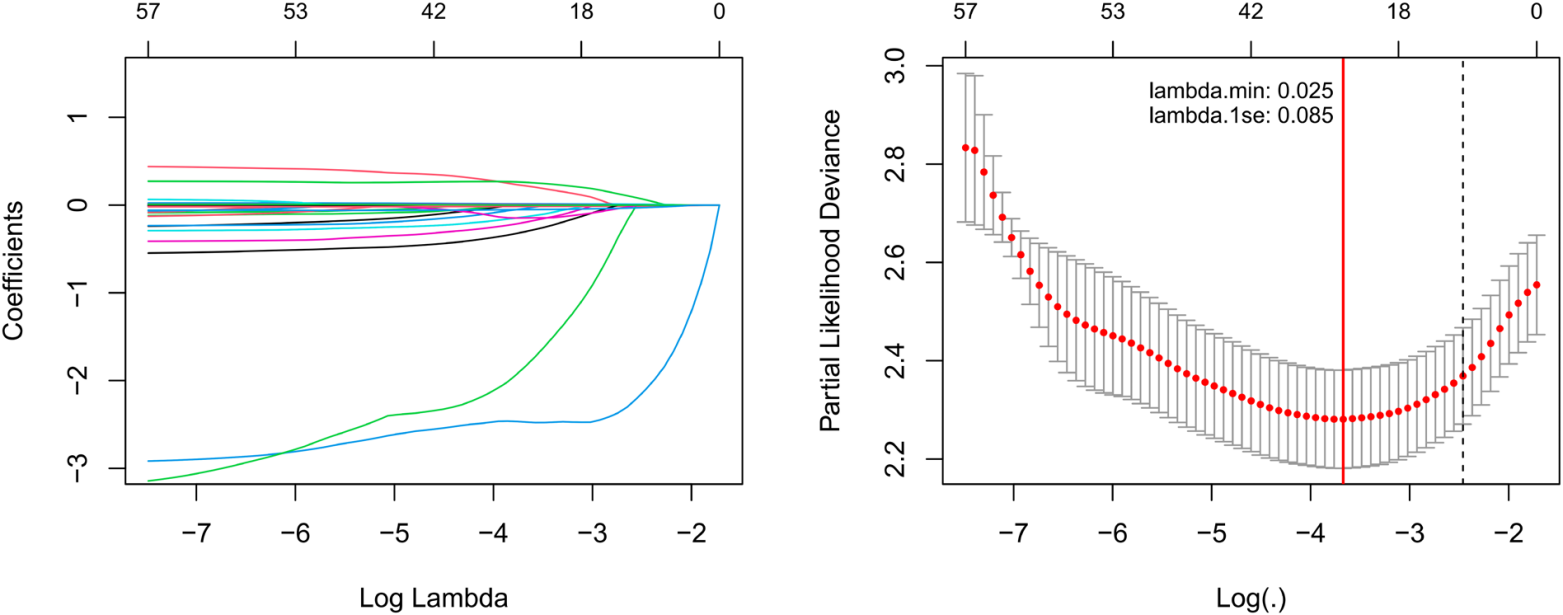
LASSO coefficient profiles and cross‐validation for optimal *λ* selection.

**TABLE 3:** Cox proportional hazards models evaluating the association between CRP–ALB joint trajectories and mortality in sepsis patients.

Outcome	Model 1		Model 2		Model 3	
	HR (95% CI)	*p*	HR (95% CI)	*p*	HR (95% CI)	*p*
**28‐day mortality**						
Trajectory group						
Slow‐decreasing	1.00 (reference)		1.00 (reference)		1.00 (reference)	
Rapid‐decreasing	1.076 (0.638, 1.812)	0.784	1.105 (0.655, 1.863)	0.708	1.201 (0.671, 2.152)	0.538
Increasing	1.764 (1.138, 2.736)	0.011	1.869 (1.200, 2.905)	0.006	1.943 (1.202, 3.140)	0.007
**60‐day mortality**						
Trajectory group						
Slow‐decreasing	1.00 (reference)		1.00 (reference)		1.00 (reference)	
Rapid‐decreasing	1.021 (0.617, 1.692)	0.934	1.059 (0.639, 1.756)	0.823	1.158 (0.663, 2.022)	0.607
Increasing	1.683 (1.098, 2.581)	0.017	1.803 (1.172, 2.774)	0.007	1.946 (1.218, 3.108)	0.005

*Note:* Model 1: unadjusted; Model 2: adjusted for age and sex; Model 3: adjusted for trajectory membership and 27 LASSO‐selected covariates.

### 3.5 Subgroup and Sensitivity Analyses

Subgroup analyses showed no evidence that the association of the increasing trajectory with mortality differed by sex, smoking, alcohol use, or infection site (all interaction *p* > 0.05) (Figure 5).

**FIGURE 5:**
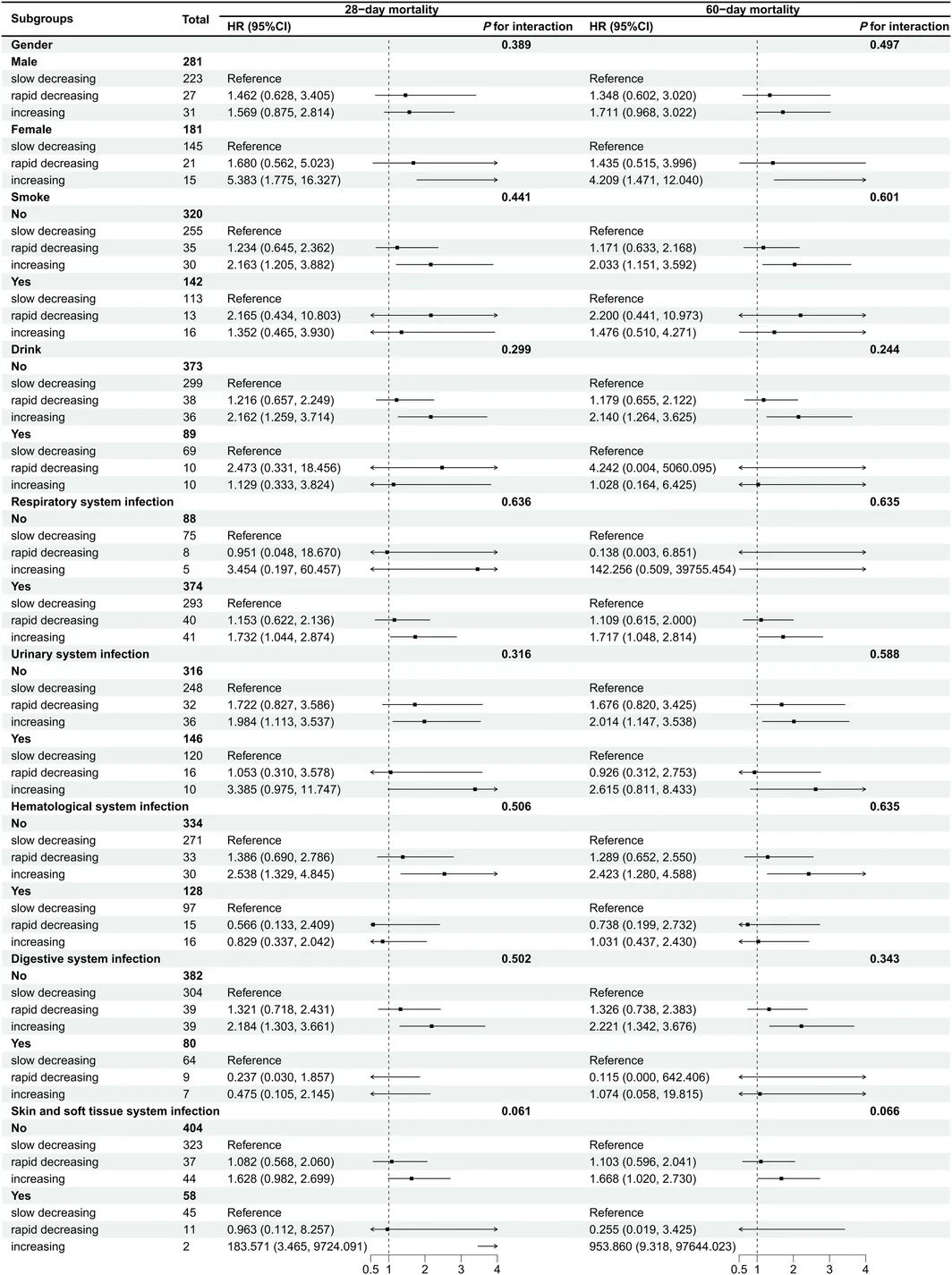
Subgroup analysis of the association between CRP–ALB joint trajectories and prognosis.

In additional fully adjusted comparisons, the CRP‐only, ALB‐only (Figures S3 and S4 and Tables S1–S4), and joint trajectory models showed similar discrimination. For 28‐day mortality, AIC values were 1723.85, 1723.49, 1724.52 and C‐indexes were 0.814, 0.810, 0.810, respectively. For 60‐day mortality, corresponding AIC values were 1909.039, 1906.012, 1908.517 and C‐indexes were 0.799, 0.797, 0.796 (Table S5). Thus, the joint model performed better than the ALB‐only model but did not outperform the CRP‐only model, and no uniform discrimination advantage was demonstrated.

Alternative class solutions produced broadly consistent but not identical high‐risk patterns. In the two‐class solution, the high‐stable group was associated with 28‐day mortality (HR 1.867, 95% CI: 1.260–2.767; *p* = 0.002) and 60‐day mortality (HR 1.746, 95% CI: 1.189–2.564; *p* = 0.004). In the four‐class solution, the high‐increasing group was not significantly associated with either outcome, whereas the moderate inverted‐U class was associated with 28‐day mortality (HR 3.197, 95% CI: 1.654–6.177; *p* = 0.001) and 60‐day mortality (HR 3.332, 95% CI: 1.774–6.260; *p* < 0.001). The latter class included only 18 patients (3.90%), indicating unstable subclassification (Figures S5 and S6 and Tables S6 and S7).

Parsimonious models yielded estimates similar to the primary analysis. After adjustment for age, sex, and SOFA, the increasing trajectory was associated with 28‐day mortality (HR 1.808, 95% CI: 1.164–2.809; *p* = 0.008) and 60‐day mortality (HR 1.745, 95% CI: 1.136–2.681; *p* = 0.011). Corresponding APACHE II‐adjusted estimates were HR 1.606 (95% CI: 1.027–2.511; *p* = 0.038) and HR 1.564 (95% CI: 1.012–2.417; *p* = 0.044) (Table S8).

Adding trajectory membership to the SOFA‐based model increased the C‐index from 0.689 to 0.702 for 28‐day mortality (Δ = 0.013; *p* = 0.055) and from 0.687 to 0.696 for 60‐day mortality (Δ = 0.009; *p* = 0.081). In APACHE II‐based models, the corresponding changes were 0.703 to 0.710 (Δ = 0.007; *p* = 0.160) and 0.697 to 0.703 (Δ = 0.006; *p* = 0.163). None reached statistical significance (Table S9).

RBC and haemoglobin had elevated VIFs in the original fully adjusted models. After RBC was excluded, all remaining VIFs were below 5, and the increasing trajectory remained associated with 28‐day mortality (HR 1.998, 95% CI: 1.247–3.202; *p* = 0.004) and 60‐day mortality (HR 1.900, 95% CI: 1.201–3.007; *p* = 0.006) (Tables S11–S13).

## 4 Discussion

In this cohort of 462 patients with sepsis, GBDTM identified three clinically interpretable joint CRP‐ALB phenotypes. The increasing phenotype was associated with both 28‐day and 60‐day mortality after multivariable adjustment. However, adding trajectory membership to models containing SOFA or APACHE II produced only small, statistically nonsignificant gains in discrimination. The principal contribution of the joint trajectory in this cohort is therefore dynamic phenotyping rather than a demonstrated replacement for established severity scores.

Most prognostic studies in sepsis rely on cross‐sectional measurements or separate single‐marker trajectories. By using repeated measurements at prespecified time points, GBDTM can describe coordinated nonlinear changes in two biologically related markers. Nevertheless, the additional model‐performance comparison showed that the joint model did not consistently outperform the CRP‐only model. Its value should therefore be interpreted as phenotypic and hypothesis‐generating rather than as evidence of superior prediction.

The observed patterns are biologically compatible with the coupled acute‐phase behaviour of CRP and ALB. Pro‐inflammatory signalling, particularly through interleukin‐6, increases hepatic CRP production while suppressing albumin synthesis. Second, pronounced ALB depletion and severe nutritional deficits tend to manifest more prominently during the chronic phase of critical illness rather than within the acute 14‐day window examined in this study [21, 22, 23].

Longitudinal trajectories can summarize temporal response patterns that are not captured by admission values alone. In this cohort, rapid CRP decline was associated with mortality similar to the slow‐decreasing group, whereas the increasing pattern identified a smaller group with higher risk. However, we did not establish the earliest time at which class membership can be assigned reliably, evaluate decision‐curve or reclassification benefit, test trajectory‐guided treatment, or show that such treatment improves outcomes. Accordingly, the findings should be considered hypothesis‐generating and require prospective validation before clinical implementation.

The prognostic contribution of joint modelling was similar across outcome horizons. The joint model had better AIC and C‐index values than the ALB‐only model but did not outperform the CRP‐only model at either 28 or 60 days. Acute short‐term risk may be driven predominantly by CRP‐related inflammatory activity, while albumin contributes a complementary description of nutritional status. The high‐risk class was also sensitive to the number of latent classes. The four‐class solution identified a 30‐patient high‐increasing class with an attenuated association and an 18‐patient moderate inverted‐U class with a strong association but prevalence below 5%. This pattern suggests possible heterogeneity within the broader adverse phenotype, but the present sample is insufficient for stable subdivision. Larger multicenter studies are required.

Several limitations should be acknowledged. First, this was a single‐center retrospective study of 462 patients; trajectory prevalence and prognostic effects may differ across geographic regions, health‐care systems, sepsis etiologies, case mix, treatment practices and measurement schedules. External validation was not performed. Future work should test reproducibility in independent multicenter cohorts and, where sufficiently dense serial CRP and ALB measurements are available, in MIMIC‐IV and eICU. Second, imputation of missing baseline covariates may introduce uncertainty. Third, the increasing group was small (*n* = 46), producing imprecise estimates. The two‐ and four‐class analyses supported an association between adverse dynamic patterns and mortality but also showed unstable high‐risk subclassification, including an 18‐patient class below the 5% threshold.

## 5 Conclusion

Joint CRP‐ALB trajectories during the first 2 weeks of sepsis identified an increasing phenotype associated with higher 28‐day and 60‐day mortality. This pattern may complement established severity scores by describing a biologically coherent dynamic phenotype, but its incremental discrimination was limited in this cohort. Prospective multicenter validation and evaluation of real time classification and clinical utility are required before the approach can inform treatment decisions.

## Author Contributions

**Tengfei Chen:** conceptualization, methodology. **Shuwen Zhang:** conceptualization, methodology. **Maoyu Ding:** conceptualization, methodology, writing – original draft. **Yumei Yang:** software, data curation. **Qinxiang Yang:** software, data curation. **Wei Guo:** writing – review and editing. **Xiaolong Xu:** writing – review and editing. **Qingquan Liu:** writing – review and editing.

## Funding

The research was supported by the National Major Science and Technology Project (2026ZD01910103); Beijing Hospital of Traditional Chinese Medicine, Capital Medical University (Grant No. LYZD202307); National Natural Science Foundation of China (82474428; 82430117); National Multidisciplinary Innovation Team Project of Traditional Chinese Medicine (ZYYCXTD‐D‐20220); National Administration of Traditional Chinese Medicine High‐Level Key Discipline of TCM project (zyyzdxk‐2023001).

## Ethics Statement

The study protocol was approved by the Ethics Committee of Beijing Hospital of Traditional Chinese Medicine (Approval No. 2024BL02099). All methods were performed in accordance with applicable guidelines and regulations.

## Consent

The authors have nothing to report.

## Conflicts of Interest

The authors declare no conflicts of interest.

## Supporting information


**Figure S1:** Proportional hazards assumption test for 28‐day mortality risk.
**Figure S2:** Proportional hazards assumption test for 60‐day mortality risk.
**Figure S3:** Trajectories of CRP in patients with sepsis.
**Figure S4:** Trajectories of ALB in patients with sepsis.
**Figure S5:** Two‐class CRP‐ALB joint trajectory solution.
**Figure S6:** Four‐class CRP‐ALB joint trajectory solution.
**Table S1:** Comparison of fit indices for the CRP trajectory models.
**Table S2:** Cox proportional hazards models for the association between CRP trajectories and prognosis in patients with sepsis.
**Table S3:** Comparison of fit indices for the ALB trajectory models.
**Table S4:** Cox proportional hazards models for the association between ALB trajectories and prognosis in patients with sepsis.
**Table S5:** Comparison of fully adjusted single‐marker and joint trajectory models.
**Table S6:** Association between two‐class CRP‐ALB trajectories and mortality.
**Table S7:** Association between four‐class CRP‐ALB trajectories and mortality.
**Table S8:** Parsimonious‐model sensitivity analysis.
**Table S9:** Incremental discrimination beyond SOFA and APACHE II.
**Table S10:** Candidate variables considered for LASSO‐Cox regression.
**Table S11:** VIFs in the fully adjusted Cox models.
**Table S12:** VIFs after exclusion of RBC.
**Table S13:** Cox models after exclusion of RBC.
**Table S14:** Complete baseline characteristics by joint trajectory group.

## Data Availability

The data that support the findings of this study are available on request from the corresponding author. The data are not publicly available due to privacy or ethical restrictions.
