# Supplementary material for: Dynamic Co‐Evolution of C‐Reactive Protein and Albumin in Early Sepsis: A Group‐Based Dual‐Trajectory Analysis for Risk Stratification
Source: J Cell Mol Med. 2026 Oct 14;30(20):e71402. doi: 10.1111/jcmm.71402 (PMC13662834; doi:10.1111/jcmm.71402)
Supplement: Supplementary file 1 [file JCMM-30-e71402-s001.docx]

**Supplementary Figure 1. Proportional hazards assumption test for 28-day mortality risk.**

**Supplementary Figure 2. Proportional hazards assumption test for 60-day mortality risk.**

**Supplementary Figure 3. Trajectories of CRP in patients with sepsis.**

**Supplementary Figure 4. Trajectories of ALB in patients with sepsis.**

**Supplementary Figure 5. Two-class CRP-ALB joint trajectory solution.**

(A) CRP and (B) ALB trajectories for the low-decreasing and high-stable classes.

**Supplementary Figure 6. Four-class CRP-ALB joint trajectory solution.**

(A) CRP and (B) ALB trajectories for the moderate inverted-U, low-slow-decreasing, low-rapid-decreasing, and high-increasing classes.

**Supplementary Table 1. Comparison of fit indices for the CRP trajectory models**

| **Group number** | **Log likelihood** | **AIC** | **BIC** | **entropy** | **class1** | **class2** | **class3** | **class4** | **class5** | **class6** |
| --- | --- | --- | --- | --- | --- | --- | --- | --- | --- | --- |
| **1** | -11406.45 | 22826.90 | 22855.85 | 1.00 | 462/100.00 |  |  |  |  |  |
| **2** | -11347.63 | 22717.25 | 22762.74 | 0.77 | 388/83.98 | 74/16.02 |  |  |  |  |
| **3** | -11299.82 | 22629.64 | 22691.68 | 0.83 | 375/81.17 | 22/4.76 | 65/14.07 |  |  |  |
| **4** | -11279.64 | 22597.29 | 22675.86 | 0.81 | 348/75.32 | 18/3.90 | 76/16.45 | 20/4.33 |  |  |
| **5** | -11223.97 | 22493.94 | 22589.06 | 0.82 | 330/71.43 | 20/4.33 | 54/11.69 | 22/4.76 | 36/7.79 |  |
| **6** | -11240.43 | 22534.86 | 22646.52 | 0.82 | 34/7.36 | 321/69.48 | 0/0.00 | 75/16.23 | 17/3.68 | 15/3.25 |

Abbreviations: AIC, Akaike Information Criterion; BIC, Bayesian Information Criterion.

**Supplementary Table 2. Cox proportional hazards models for the association between CRP trajectories and prognosis in patients with sepsis**

| **Outcome** | **Model 1** | | **Model 2** | | **Model 3** | |
| --- | --- | --- | --- | --- | --- | --- |
|  | **HR (95%CI)** | ***P*-value** | **HR (95%CI)** | ***P*-value** | **HR (95%CI)** | ***P*-value** |
| **28-day mortality** |  |  |  |  |  |  |
| Low-decreasing | 1.00(reference) |  | 1.00(reference) |  | 1.00(reference) |  |
| High-stable | 1.934(1.352, 2.768) | <0.001 | 1.975(1.379, 2.829) | <0.001 | 1.867(1.260, 2.767) | 0.002 |
| **60-day mortality** |  |  |  |  |  |  |
| Low-decreasing | 1.00(reference) |  | 1.00(reference) |  | 1.00(reference) |  |
| High-stable | 1.807(1.273, 2.564) | 0.001 | 1.856(1.306, 2.638) | 0.001 | 1.746(1.189, 2.564) | 0.004 |

Model 1 = unadjusted; Model 2 = adjusted for age and sex; Model 3 = adjusted using the 27-covariate LASSO-derived adjustment set.

**Supplementary Table 3. Comparison of fit indices for the ALB trajectory models**

| **Group number** | **Log likelihood** | **AIC** | **BIC** | **entropy** | **class1** | **class2** | **class3** | **class4** | **class5** | **class6** |
| --- | --- | --- | --- | --- | --- | --- | --- | --- | --- | --- |
| **1** | -5453.35 | 10920.70 | 10949.65 | 1.00 | 462/100.00 |  |  |  |  |  |
| **2** | -5401.26 | 10824.52 | 10870.01 | 0.82 | 427/92.42 | 35/7.58 |  |  |  |  |
| **3** | -5389.68 | 10809.35 | 10871.38 | 0.67 | 392/84.85 | 43/9.31 | 27/5.84 |  |  |  |
| **4** | -5381.78 | 10801.56 | 10880.13 | 0.59 | 328/71.00 | 64/13.85 | 50/10.82 | 20/4.33 |  |  |
| **5** | -5373.56 | 10793.11 | 10888.23 | 0.64 | 319/69.05 | 14/3.03 | 47/10.17 | 60/12.99 | 22/4.76 |  |
| **6** | -5373.10 | 10800.21 | 10911.87 | 0.57 | 27/5.84 | 61/13.20 | 275/59.52 | 56/12.12 | 26/5.63 | 17/3.68 |

Abbreviations: AIC, Akaike Information Criterion; BIC, Bayesian Information Criterion.

**Supplementary Table 4. Cox proportional hazards models for the association between ALB trajectories and prognosis in patients with sepsis**

| **Outcome** | **Model 1** | | **Model 2** | | **Model 3** | |
| --- | --- | --- | --- | --- | --- | --- |
|  | **HR (95%CI)** | ***P*-value** | **HR (95%CI)** | ***P*-value** | **HR (95%CI)** | ***P*-value** |
| **28-day mortality** |  |  |  |  |  |  |
| Low-increasing | 1.00(reference) |  | 1.00(reference) |  | 1.00(reference) |  |
| High-increasing | 0.344(0.141, 0.837) | 0.019 | 0.376(0.153, 0.920) | 0.032 | 0.387(0.154, 0.975) | 0.044 |
| **60-day mortality** |  |  |  |  |  |  |
| Low-increasing | 1.00(reference) |  | 1.00(reference) |  | 1.00(reference) |  |
| High-increasing | 0.306(0.126, 0.744) | 0.009 | 0.341(0.140, 0.834) | 0.018 | 0.357(0.138, 0.925) | 0.034 |

Model 1 = unadjusted; Model 2 = adjusted for age and sex; Model 3 = adjusted using the 27-covariate LASSO-derived adjustment set.

**Supplementary Table 5. Comparison of fully adjusted single-marker and joint trajectory models**

|  |  | Model 1 (CRP-only) | Model 2 (ALB-only) | Model 3 (CRP-ALB) | Model 1vs 3 LRT‑P | Model 2 vs 3 LRT‑P |
| --- | --- | --- | --- | --- | --- | --- |
| 28-day mortality | AIC | 1723.85 | 1723.49 | 1724.52 | - | - |
|  | Harrell C-index | 0.814 | 0.810 | 0.810 | - | - |
|  | LRT P‑value | - | - | - | 0.102 | 0.082 |
| 60-day mortality | AIC | 1909.039 | 1906.012 | 1908.517 | - | - |
|  | Harrell C-index | 0.799 | 0.797 | 0.796 | - | - |
|  | LRT P‑value | - | - | - | 0.224 | 0.034 |

Model 1: CRP-only trajectory exposure plus the same 27-covariate adjustment set.

Model 2: ALB-only trajectory exposure plus the same 27-covariate adjustment set.

Model 3: CRP-ALB joint trajectory exposure plus the same 27-covariate adjustment set.

Because the latent-class models were non-nested, AIC and Harrell C-index were compared descriptively; pairwise likelihood-ratio tests were not performed.

**Supplementary Table 6. Association between two-class CRP-ALB trajectories and mortality**

| **Outcome** | **Model 1** | | **Model 2** | | **Model 3** | |
| --- | --- | --- | --- | --- | --- | --- |
|  | **HR (95% CI)** | **P value** | **HR (95% CI)** | **P value** | **HR (95% CI)** | **P value** |
| **28-day mortality** |  |  |  |  |  |  |
| **Trajectory group** |  |  |  |  |  |  |
| Low-decreasing | 1.000 (reference) |  | 1.000 (reference) |  | 1.000 (reference) |  |
| High-stable | 1.934 (1.352-2.768) | <0.001 | 1.975 (1.379-2.829) | <0.001 | 1.867 (1.260-2.767) | 0.002 |
| **60-day mortality** |  |  |  |  |  |  |
| **Trajectory group** |  |  |  |  |  |  |
| Low-decreasing | 1.000 (reference) |  | 1.000 (reference) |  | 1.000 (reference) |  |
| High-stable | 1.807 (1.273-2.564) | 0.001 | 1.856 (1.306-2.638) | 0.001 | 1.746 (1.189-2.564) | 0.004 |

Model 1: unadjusted.

Model 2: adjusted for age and sex.

Model 3: trajectory membership plus the same 27-covariate LASSO-derived adjustment set.

**Supplementary Table 7. Association between four-class CRP-ALB trajectories and mortality**

| **Outcome** | **Model 1** | | **Model 2** | | **Model 3** | |
| --- | --- | --- | --- | --- | --- | --- |
|  | **HR (95% CI)** | **P value** | **HR (95% CI)** | **P value** | **HR (95% CI)** | **P value** |
| **28-day mortality** |  |  |  |  |  |  |
| **Trajectory group** |  |  |  |  |  |  |
| Low-slow-decreasing | 1.000 (reference) |  | 1.000 (reference) |  | 1.000 (reference) |  |
| Low-rapid-decreasing | 1.064 (0.639-1.769) | 0.812 | 1.099 (0.661-1.830) | 0.715 | 1.124 (0.643-1.966) | 0.682 |
| High-increasing | 1.303 (0.748-2.268) | 0.350 | 1.304 (0.749-2.270) | 0.349 | 1.212 (0.669-2.197) | 0.526 |
| Moderate inverted-U | 3.195 (1.759-5.803) | <0.001 | 3.380 (1.855-6.160) | <0.001 | 3.197 (1.654-6.177) | 0.001 |
| **60-day mortality** |  |  |  |  |  |  |
| **Trajectory group** |  |  |  |  |  |  |
| Low-slow-decreasing | 1.000 (reference) |  | 1.000 (reference) |  | 1.000 (reference) |  |
| Low-rapid-decreasing | 1.003 (0.613-1.641) | 0.991 | 1.047 (0.639-1.716) | 0.855 | 1.067 (0.621-1.832) | 0.815 |
| High-increasing | 1.193 (0.687-2.070) | 0.531 | 1.198 (0.690-2.080) | 0.520 | 1.134 (0.631-2.040) | 0.674 |
| Moderate inverted-U | 3.195 (1.802-5.666) | <0.001 | 3.409 (1.917-6.063) | <0.001 | 3.332 (1.774-6.260) | <0.001 |

Model 1: unadjusted.

Model 2: adjusted for age and sex.

Model 3: trajectory membership plus the same 27-covariate LASSO-derived adjustment set.

**Supplementary Table 8. Parsimonious-model sensitivity analysis**

| **Outcome** | **Simplified model 1** | | **Simplified model 2** | | **Fully adjusted model** | |
| --- | --- | --- | --- | --- | --- | --- |
|  | **HR (95% CI)** | **P value** | **HR (95% CI)** | **P value** | **HR (95% CI)** | **P value** |
| **28-day mortality** |  |  |  |  |  |  |
| **Trajectory group** |  |  |  |  |  |  |
| Slow-decreasing | 1.000 (reference) |  | 1.000 (reference) |  | 1.000 (reference) |  |
| Rapid-decreasing | 0.897 (0.531-1.514) | 0.683 | 0.864 (0.511-1.460) | 0.585 | 1.201 (0.671-2.152) | 0.538 |
| Increasing | 1.808 (1.164-2.809) | 0.008 | 1.606 (1.027-2.511) | 0.038 | 1.943 (1.202-3.140) | 0.007 |
| **60-day mortality** |  |  |  |  |  |  |
| **Trajectory group** |  |  |  |  |  |  |
| Slow-decreasing | 1.000 (reference) |  | 1.000 (reference) |  | 1.000 (reference) |  |
| Rapid-decreasing | 0.861 (0.519-1.429) | 0.562 | 0.837 (0.504-1.391) | 0.492 | 1.158 (0.663-2.022) | 0.607 |
| Increasing | 1.745 (1.136-2.681) | 0.011 | 1.564 (1.012-2.417) | 0.044 | 1.946 (1.218-3.108) | 0.005 |

Simplified model 1: adjusted for age, sex, and SOFA.

Simplified model 2: adjusted for age, sex, and APACHE II.

Fully adjusted model: trajectory membership plus the same 27-covariate LASSO-derived adjustment set.

SOFA and APACHE II were fitted in separate models because they represent overlapping severity constructs.

**Supplementary Table 9. Incremental discrimination beyond SOFA and APACHE II**

| **Outcome** | **Model** | **C-index** | **Delta C-index** | **P value** |
| --- | --- | --- | --- | --- |
| 28-day mortality | Model 1 | 0.689 | - | - |
|  | Model 2 | 0.702 | 0.013 | 0.055 |
|  | Model 3 | 0.703 | - | - |
|  | Model 4 | 0.710 | 0.007 | 0.160 |
| 60-day mortality | Model 1 | 0.687 | - | - |
|  | Model 2 | 0.696 | 0.009 | 0.081 |
|  | Model 3 | 0.697 | - | - |
|  | Model 4 | 0.703 | 0.006 | 0.163 |

Model 1: adjusted for age, sex, and SOFA.

Model 2: adjusted for age, sex, SOFA, and CRP-ALB joint trajectory membership.

Model 3: adjusted for age, sex, and APACHE II.

Model 4: adjusted for age, sex, APACHE II, and CRP-ALB joint trajectory membership.

Delta C-index is the difference between the model with and without CRP-ALB joint trajectory membership.

**Supplementary Table 10. Candidate variables considered for LASSO-Cox regression**

| **Category** | **Candidate variables** | **Rationale** |
| --- | --- | --- |
| Demographics | Age; sex | Established baseline confounders for ICU mortality |
| Vital signs | Temperature; heart rate; respiratory rate; SBP; DBP; MAP | Acute physiological disturbance |
| Infection sites | Respiratory; urinary; hematological; digestive; skin and soft tissue | Source of infection may influence prognosis |
| Habits | Smoking; alcohol use | Potential modifiers of host response and outcome |
| Organ function and laboratory | WBC; NEU; RBC; Hgb; Hct; PLT; PCT; PT; APTT; FIB; TT; D-dimer; FDP; ALT; AST; TB; DB; ALP; GGT; LDH; BUN; creatinine; eGFR; uric acid; pH; PaO2; BNP; troponin I; CK; CK-MB; AGI grade; potassium; sodium; bile acids; myoglobin | Inflammation, coagulation, organ injury, electrolyte status, and hematological function |
| ICU treatment and stay | ICU stay; hospital stay; invasive ventilation; noninvasive ventilation; hemofiltration | Intensity and duration of critical-care support |
| Echocardiography | LAAPD; LVEDD; LVEF | Cardiac structure and function |
| Neurological | GCS | Neurological status |

**Supplementary Table 11. VIFs in the fully adjusted Cox models**

| **Variable** | **28-day VIF** | **60-day VIF** |
| --- | --- | --- |
| Age | 1.16 | 1.16 |
| Sex | 1.12 | 1.12 |
| Heart rate | 1.18 | 1.16 |
| Respiratory infection | 1.55 | 1.52 |
| Hematological infection | 1.24 | 1.22 |
| Digestive infection | 1.51 | 1.47 |
| Skin/soft-tissue infection | 1.22 | 1.22 |
| ICU stay | 1.18 | 1.22 |
| Hospital stay | 1.20 | 1.25 |
| Invasive ventilation time | 1.21 | 1.23 |
| MAP | 1.38 | 1.34 |
| AGI | 1.19 | 1.19 |
| pH | 1.56 | 1.46 |
| PaO2 | 1.21 | 1.20 |
| Total bilirubin | 1.22 | 1.22 |
| Bile acids | 1.08 | 1.08 |
| APTT | 1.19 | 1.19 |
| FDP | 1.20 | 1.20 |
| BUN | 1.28 | 1.28 |
| BNP | 1.25 | 1.24 |
| CK-MB | 1.24 | 1.24 |
| LAAPD | 1.27 | 1.29 |
| LVEF | 1.15 | 1.18 |
| RBC | 8.24 | 8.88 |
| Hemoglobin | 8.31 | 9.00 |
| PCT | 1.25 | 1.24 |
| GCS | 1.22 | 1.21 |

**Supplementary Table 12. VIFs after exclusion of RBC**

| **Variable** | **28-day VIF** | **60-day VIF** |
| --- | --- | --- |
| Age | 1.16 | 1.16 |
| Sex | 1.11 | 1.11 |
| Heart rate | 1.17 | 1.15 |
| Respiratory infection | 1.55 | 1.52 |
| Hematological infection | 1.24 | 1.22 |
| Digestive infection | 1.51 | 1.47 |
| Skin/soft-tissue infection | 1.22 | 1.21 |
| ICU stay | 1.17 | 1.21 |
| Hospital stay | 1.20 | 1.24 |
| Invasive ventilation time | 1.21 | 1.22 |
| MAP | 1.37 | 1.33 |
| AGI | 1.19 | 1.19 |
| pH | 1.53 | 1.44 |
| PaO2 | 1.21 | 1.20 |
| Total bilirubin | 1.22 | 1.22 |
| Bile acids | 1.08 | 1.08 |
| APTT | 1.19 | 1.18 |
| FDP | 1.20 | 1.19 |
| BUN | 1.28 | 1.27 |
| BNP | 1.25 | 1.24 |
| CK-MB | 1.24 | 1.23 |
| LAAPD | 1.27 | 1.29 |
| LVEF | 1.14 | 1.16 |
| Hemoglobin | 1.20 | 1.20 |
| PCT | 1.25 | 1.23 |
| GCS | 1.22 | 1.21 |

**Supplementary Table 13. Cox models after exclusion of RBC**

| **Outcome** | **Trajectory group** | **HR (95% CI)** | **P value** |
| --- | --- | --- | --- |
| 28-day | Slow-decreasing | 1.000 (reference) |  |
| 28-day | Rapid-decreasing | 1.141 (0.646-2.016) | 0.650 |
| 28-day | Increasing | 1.998 (1.247-3.202) | 0.004 |
| 60-day | Slow-decreasing | 1.000 (reference) |  |
| 60-day | Rapid-decreasing | 1.041 (0.601-1.802) | 0.887 |
| 60-day | Increasing | 1.900 (1.201-3.007) | 0.006 |

**Supplementary Table 14. Complete baseline characteristics by joint trajectory group**

| **Characteristic** | **Total (n = 462)** | **Slow-decreasing (n = 368)** | **Rapid-decreasing (n = 48)** | **Increasing (n = 46)** | **P value** |
| --- | --- | --- | --- | --- | --- |
| Gender, n(%) |  |  |  |  | 0.532 |
| Male | 281 (60.8) | 223 (60.6) | 27 (56.2) | 31 (67.4) |  |
| Female | 181 (39.2) | 145 (39.4) | 21 (43.8) | 15 (32.6) |  |
| Age (year) (mean (SD)) | 76.29 (13.54) | 76.80 (13.69) | 76.25 (10.22) | 72.26 (14.93) | 0.100 |
| Body temperature (℃), M (Q₁, Q₃) | 36.80 (36.42, 37.50) | 36.70 (36.40, 37.40) | 37.20 (36.60, 37.92) | 36.95 (36.60, 37.70) | 0.022 |
| Heart rate (/Min), M (Q₁, Q₃) | 90.50 (78.00, 108.00) | 90.50 (78.00, 108.00) | 93.00 (82.00, 111.00) | 89.00 (83.00, 106.75) | 0.285 |
| Respiratory rate (/Min), M (Q₁, Q₃) | 20.00 (18.00, 25.00) | 20.00 (18.00, 25.00) | 21.00 (18.00, 25.25) | 22.00 (19.00, 25.75) | 0.595 |
| SBP (mmHg), M (Q₁, Q₃) | 126.00 (110.00, 140.00) | 126.00 (110.00, 141.25) | 121.00 (106.75, 136.25) | 130.00 (118.00, 138.50) | 0.340 |
| DBP (mmHg) (mean (SD)) | 71.24 (15.71) | 71.70 (16.00) | 68.27 (13.73) | 70.59 (15.20) | 0.348 |
| Respiratory system infection , n(%) |  |  |  |  | 0.273 |
| No | 88 (19.0) | 75 (20.4) | 8 (16.7) | 5 (10.9) |  |
| Yes | 374 (81.0) | 293 (79.6) | 40 (83.3) | 41 (89.1) |  |
| Urinary system infection , n(%) |  |  |  |  | 0.315 |
| No | 316 (68.4) | 248 (67.4) | 32 (66.7) | 36 (78.3) |  |
| Yes | 146 (31.6) | 120 (32.6) | 16 (33.3) | 10 (21.7) |  |
| Hematological system infection, n(%) |  |  |  |  | 0.410 |
| No | 334 (72.3) | 271 (73.6) | 33 (68.8) | 30 (65.2) |  |
| Yes | 128 (27.7) | 97 (26.4) | 15 (31.2) | 16 (34.8) |  |
| Digestive system infection , n(%) |  |  |  |  | 0.899 |
| No | 382 (82.7) | 304 (82.6) | 39 (81.2) | 39 (84.8) |  |
| Yes | 80 (17.3) | 64 (17.4) | 9 (18.8) | 7 (15.2) |  |
| Infection of the skin and soft tissues, n(%) |  |  |  |  | 0.023 |
| No | 404 (87.4) | 323 (87.8) | 37 (77.1) | 44 (95.7) |  |
| Yes | 58 (12.6) | 45 (12.2) | 11 (22.9) | 2 ( 4.3) |  |
| Smoke, n(%) |  |  |  |  | 0.721 |
| No | 320 (69.3) | 255 (69.3) | 35 (72.9) | 30 (65.2) |  |
| Yes | 142 (30.7) | 113 (30.7) | 13 (27.1) | 16 (34.8) |  |
| Drink, n(%) |  |  |  |  | 0.852 |
| No | 373 (80.7) | 299 (81.2) | 38 (79.2) | 36 (78.3) |  |
| Yes | 89 (19.3) | 69 (18.8) | 10 (20.8) | 10 (21.7) |  |
| ICU stay (day), M (Q₁, Q₃) | 14.00 (7.00, 22.00) | 14.00 (7.00, 22.00) | 11.00 (6.75, 20.00) | 14.00 (4.00, 22.50) | 0.632 |
| Hospital stay (day), M (Q₁, Q₃) | 23.00 (12.00, 41.00) | 24.00 (12.00, 41.00) | 19.00 (10.75, 46.00) | 23.50 (13.25, 39.75) | 0.798 |
| Invasive ventilator time (h), M (Q₁, Q₃) | 9.00 (0.00, 149.75) | 9.50 (0.00, 147.25) | 18.00 (0.00, 122.25) | 8.50 (0.00, 178.50) | 0.942 |
| Non-invasive ventilator time (h), M (Q₁, Q₃) | 3.50 (0.00, 109.50) | 1.50 (0.00, 108.50) | 0.50 (0.00, 92.75) | 35.00 (0.00, 149.25) | 0.321 |
| Hemofiltration (h), M (Q₁, Q₃) | 0.00 (0.00, 14.00) | 0.00 (0.00, 1.25) | 0.00 (0.00, 51.75) | 0.00 (0.00, 89.25) | 0.019 |
| MAP (mmHg), M (Q₁, Q₃) | 87.00 (70.42, 98.00) | 87.00 (72.00, 100.00) | 79.00 (67.75, 98.00) | 86.00 (74.00, 93.50) | 0.397 |
| AGI, n(%) |  |  |  |  | 0.015 |
| 0 | 5 ( 1.1) | 3 ( 0.8) | 2 ( 4.2) | 0 ( 0.0) |  |
| 1 | 11 ( 2.4) | 6 ( 1.6) | 1 ( 2.1) | 4 ( 8.7) |  |
| 2 | 29 ( 6.3) | 26 ( 7.1) | 1 ( 2.1) | 2 ( 4.3) |  |
| 3 | 140 (30.3) | 106 (28.8) | 20 (41.7) | 14 (30.4) |  |
| 4 | 277 (60.0) | 227 (61.7) | 24 (50.0) | 26 (56.5) |  |
| pH, M (Q₁, Q₃) | 7.43 (7.34, 7.47) | 7.43 (7.34, 7.47) | 7.40 (7.34, 7.46) | 7.45 (7.38, 7.49) | 0.036 |
| PaO2 (mmHg), M (Q₁, Q₃) | 108.00 (80.00, 143.00) | 105.00 (80.00, 140.00) | 115.50 (80.22, 158.25) | 123.50 (80.30, 146.00) | 0.390 |
| DB (μmol/L), M (Q₁, Q₃) | 7.05 (3.62, 13.20) | 6.65 (3.60, 13.20) | 10.60 (5.05, 13.20) | 7.75 (3.90, 13.20) | 0.249 |
| TB (μmol/L), M (Q₁, Q₃) | 14.95 (8.72, 26.70) | 14.70 (8.70, 26.70) | 19.20 (10.23, 26.70) | 18.45 (8.38, 26.02) | 0.651 |
| Bile acids (μmol/L), M (Q₁, Q₃) | 5.40 (2.90, 10.90) | 5.30 (2.88, 10.90) | 6.85 (3.15, 10.90) | 5.70 (3.15, 10.90) | 0.831 |
| ALT (U/L), M (Q₁, Q₃) | 21.60 (11.90, 47.50) | 22.45 (12.00, 47.50) | 21.45 (13.65, 57.95) | 17.35 (9.08, 39.40) | 0.381 |
| AST (U/L), M (Q₁, Q₃) | 35.05 (20.35, 72.70) | 36.15 (21.03, 73.18) | 33.00 (19.70, 62.33) | 35.20 (19.30, 59.43) | 0.647 |
| GGT (U/L), M (Q₁, Q₃) | 38.10 (22.63, 74.12) | 35.05 (22.40, 73.55) | 44.45 (20.32, 67.35) | 40.70 (29.45, 106.18) | 0.279 |
| ALP (U/L), M (Q₁, Q₃) | 88.10 (64.05, 133.30) | 86.60 (64.00, 134.45) | 92.25 (64.68, 121.87) | 99.25 (67.80, 133.30) | 0.615 |
| LDH (U/L), M (Q₁, Q₃) | 295.70 (220.88, 421.50) | 292.40 (212.82, 421.02) | 291.00 (246.75, 359.20) | 334.50 (257.62, 477.85) | 0.291 |
| MB (μg /L), M (Q₁, Q₃) | 174.50 (76.78, 453.95) | 166.00 (72.72, 456.50) | 204.05 (114.43, 527.90) | 204.50 (83.55, 395.05) | 0.334 |
| PT (s), M (Q₁, Q₃) | 13.60 (12.20, 15.70) | 13.60 (12.20, 15.70) | 13.70 (13.02, 16.05) | 13.80 (12.15, 15.50) | 0.387 |
| FIB (g/L) (mean (SD)) | 3.90 (1.42) | 3.73 (1.37) | 5.06 (1.27) | 4.12 (1.31) | <0.001 |
| APTT (s), M (Q₁, Q₃) | 33.10 (29.22, 38.68) | 33.25 (29.30, 38.80) | 32.30 (29.05, 35.78) | 34.30 (29.75, 39.33) | 0.490 |
| TT (s), M (Q₁, Q₃) | 14.70 (13.40, 16.30) | 14.90 (13.50, 16.50) | 14.00 (13.05, 15.05) | 14.60 (13.07, 16.10) | 0.008 |
| DD (mg/L), M (Q₁, Q₃) | 1.60 (0.80, 3.27) | 1.50 (0.80, 3.20) | 2.20 (1.00, 3.62) | 1.60 (1.02, 3.15) | 0.259 |
| FDP (mg/L), M (Q₁, Q₃) | 10.65 (6.12, 21.65) | 10.55 (6.00, 21.22) | 13.50 (6.38, 22.68) | 10.10 (6.72, 23.28) | 0.477 |
| CK (U/L), M (Q₁, Q₃) | 92.95 (40.92, 255.57) | 92.20 (39.58, 271.20) | 94.00 (49.80, 212.07) | 98.40 (33.38, 197.52) | 0.922 |
| BUN (mmol/L), M (Q₁, Q₃) | 11.30 (6.43, 20.92) | 10.60 (6.38, 19.55) | 15.90 (7.20, 24.13) | 13.20 (8.32, 23.05) | 0.104 |
| Cr (μmol/L), M (Q₁, Q₃) | 92.30 (61.00, 183.65) | 91.15 (59.70, 180.92) | 129.40 (67.40, 213.35) | 97.80 (72.18, 181.05) | 0.219 |
| eGFR (ml/min), M (Q₁, Q₃) | 61.15 (25.72, 89.18) | 62.10 (27.55, 89.23) | 44.00 (21.60, 84.40) | 54.00 (26.62, 89.20) | 0.361 |
| UA (mmol/L), M (Q₁, Q₃) | 272.40 (170.82, 406.45) | 271.35 (170.45, 405.28) | 284.85 (200.78, 501.70) | 268.55 (169.32, 401.85) | 0.548 |
| Blood potassium (mmol/L), M (Q₁, Q₃) | 4.10 (3.70, 4.60) | 4.10 (3.70, 4.60) | 4.10 (3.70, 4.50) | 3.95 (3.52, 4.68) | 0.757 |
| Blood sodium (mmol/L), M (Q₁, Q₃) | 138.30 (134.72, 143.95) | 138.35 (134.80, 143.70) | 136.85 (134.40, 142.48) | 138.70 (135.38, 145.38) | 0.720 |
| BNP (ng/L), M (Q₁, Q₃) | 557.50 (227.00, 1799.67) | 539.50 (233.00, 1884.25) | 593.00 (198.50, 1557.75) | 670.50 (365.75, 1783.00) | 0.790 |
| TNI (pg/ml), M (Q₁, Q₃) | 8.11 (0.05, 80.00) | 5.60 (0.05, 78.73) | 22.33 (0.05, 144.36) | 12.29 (0.05, 62.60) | 0.240 |
| CKMB (ng/ml), M (Q₁, Q₃) | 2.90 (1.20, 8.90) | 2.60 (1.10, 9.57) | 4.55 (1.37, 8.45) | 3.95 (1.92, 8.67) | 0.211 |
| LAAPD (cm), M (Q₁, Q₃) | 3.50 (3.20, 3.80) | 3.50 (3.20, 3.80) | 3.45 (3.30, 3.70) | 3.45 (3.20, 3.80) | 0.871 |
| LVEDD (cm), M (Q₁, Q₃) | 4.60 (4.32, 5.00) | 4.70 (4.40, 5.00) | 4.60 (4.20, 5.00) | 4.60 (4.30, 5.07) | 0.646 |
| LVEF, M (Q₁, Q₃) | 0.61 (0.58, 0.63) | 0.60 (0.58, 0.63) | 0.62 (0.59, 0.65) | 0.60 (0.58, 0.63) | 0.409 |
| WBC (*109/L), M (Q₁, Q₃) | 11.50 (7.80, 16.67) | 11.15 (7.60, 16.42) | 14.60 (9.88, 20.73) | 11.70 (8.77, 16.12) | 0.029 |
| NEU (*109/L), M (Q₁, Q₃) | 10.10 (6.30, 14.67) | 9.80 (6.18, 14.10) | 13.10 (8.35, 18.77) | 10.70 (7.70, 14.45) | 0.007 |
| RBC (*1012/L) (mean (SD)) | 3.17 (0.87) | 3.18 (0.87) | 3.21 (0.84) | 3.05 (0.90) | 0.596 |
| Hgb (g/L) (mean (SD)) | 94.56 (26.26) | 94.91 (25.63) | 96.10 (29.62) | 90.15 (27.71) | 0.466 |
| Hct (%) (mean (SD)) | 29.01 (7.96) | 29.06 (7.88) | 29.98 (8.11) | 27.55 (8.42) | 0.323 |
| GCS, M (Q₁, Q₃) | 6.00 (3.00, 14.00) | 6.50 (3.00, 14.00) | 6.00 (3.00, 13.25) | 7.50 (3.00, 15.00) | 0.713 |
| Follow-up time (28d) (mean (SD)) | 21.20 (10.26) | 21.69 (10.06) | 20.56 (11.10) | 17.98 (10.62) | 0.062 |
| Outcome 28day, n(%) |  |  |  |  | 0.031 |
| 0 = Life | 302 (65.4) | 248 (67.4) | 32 (66.7) | 22 (47.8) |  |
| 1 = Death | 160 (34.6) | 120 (32.6) | 16 (33.3) | 24 (52.2) |  |
| Follow-up time (60 d) (mean (SD)) | 41.92 (24.81) | 43.17 (24.33) | 41.33 (25.96) | 32.59 (25.92) | 0.024 |
| Outcome 60day, n(%) |  |  |  |  |  |
| 0 = Life | 286 (61.9) | 234 (63.6) | 31 (64.6) | 21 (45.7) | 0.057 |
| 1 = Death | 176 (38.1) | 134 (36.4) | 17 (35.4) | 25 (54.3) |  |
